# Phylogenetic Structure and Comparative Genomics of Multi-National Invasive *Haemophilus influenzae* Serotype a Isolates

**DOI:** 10.3389/fmicb.2022.856884

**Published:** 2022-03-24

**Authors:** Nadav Topaz, Raymond Tsang, Ala-Eddine Deghmane, Heike Claus, Thiên-Trí Lâm, David Litt, Maria Paula Bajanca-Lavado, María Pérez-Vázquez, Didrik Vestrheim, Maria Giufrè, Arie Van Der Ende, Olivier Gaillot, Alicja Kuch, Martha McElligott, Muhamed-Kheir Taha, Xin Wang

**Affiliations:** ^1^Meningitis and Vaccine Preventable Diseases Branch, Division of Bacterial Diseases, National Center for Immunization and Respiratory Diseases, Centers for Disease Control and Prevention, Atlanta, GA, United States; ^2^Vaccine Preventable Bacterial Diseases, National Microbiology Laboratory, Public Health Agency of Canada, Winnipeg, MB, Canada; ^3^Centre National de Référence des Méningocoques, Institut Pasteur, Paris, France; ^4^Institute for Hygiene and Microbiology, University of Würzburg, Würzburg, Germany; ^5^Respiratory and Vaccine Preventable Bacterial Reference Unit, Public Health England, London, United Kingdom; ^6^Haemophilus Influenzae Reference Laboratory, Department of Infectious Disease, National Institute of Health, Lisbon, Portugal; ^7^Laboratorio de Referencia e Investigación en Resistencia a Antibióticos e Infecciones Relacionadas con la Asistencia Sanitaria, Centro Nacional de Microbiología, Instituto de Salud Carlos III, Madrid, Spain; ^8^Norwegian Institute of Public Health, Division of Infection Control and Environmental Health, Oslo, Norway; ^9^Department of Infectious Diseases, Istituto Superiore di Sanità, Rome, Italy; ^10^Department of Medical Microbiology and Infection Prevention and the Netherlands Reference Laboratory for Bacterial Meningitis, University of Amsterdam, Amsterdam, Netherlands; ^11^Service de Bactériologie-Hygiène, CHU Lille, Lille, France; ^12^CNRS, INSERM, U1019-UMR 8204, Center for Infection and Immunity, CHU Lille, Lille, France; ^13^Department of Epidemiology and Clinical Microbiology, National Medicines Institute, Warsaw, Poland; ^14^Irish Meningitis and Sepsis Reference Laboratory, Children’s Health Ireland at Temple Street, Dublin, Ireland; ^15^Meningitis and Vaccine Preventable Diseases Branch, Division of Bacterial Diseases, National Center for Immunization and Respiratory Diseases, Centers for Disease Control and Prevention, Atlanta, GA, United States

**Keywords:** serotype a, genomics, phylogenetic analysis, invasive isolates, *Haemophilus influenzae*

## Abstract

Recent reports have indicated a rise of invasive disease caused by *Haemophilus influenzae* serotype a (Hia) in North America and some European countries. The whole-genome sequences for a total of 410 invasive Hia isolates were obtained from 12 countries spanning the years of 1998 to 2019 and underwent phylogenetic and comparative genomic analysis in order to characterize the major strains causing disease and the genetic variation present among factors contributing to virulence and antimicrobial resistance. Among 410 isolate sequences received, 408 passed our quality control and underwent genomic analysis. Phylogenetic analysis revealed that the Hia isolates formed four genetically distinct clades: clade 1 (*n* = 336), clade 2 (*n* = 13), clade 3 (*n* = 3) and clade 4 (*n* = 56). A low diversity subclade 1.1 was found in clade 1 and contained almost exclusively North American isolates. The predominant sequence types in the Hia collection were ST-56 (*n* = 125), ST-23 (*n* = 98) and ST-576 (*n* = 51), which belonged to clade 1, and ST-62 (*n* = 54), which belonged to clade 4. Clades 1 and 4 contained predominantly North American isolates, and clades 2 and 3 predominantly contained European isolates. Evidence of the presence of capsule duplication was detected in clade 1 and 2 isolates. Seven of the virulence genes involved in endotoxin biosynthesis were absent from all Hia isolates. In general, the presence of known factors contributing to β-lactam antibiotic resistance was low among Hia isolates. Further tests for virulence and antibiotic susceptibility would be required to determine the impact of these variations among the isolates.

## Introduction

*Haemophilus influenzae* (*H. influenzae*) is a gram negative, commensal organism found in the human respiratory tract that can occasionally cause serious disease such as meningitis, pneumonia and bacteremia (Turk, 1984). There are six encapsulated serotypes (Hia, Hib, Hic, Hid, Hie and Hif), as well as unencapsulated *H. influenzae*, referred to as non-typeable *Haemophilus influenzae* (NTHi). Isolates of each encapsulated serotype produce a unique polysaccharide capsule encoded by the *H. influenzae* capsule locus (Potts et al., 2019), whereas NTHi do not possess this gene locus.

Historically, Hib was the primary cause of meningitis in children under 5 years of age, as well as a major cause of other serious infections such as pneumonia and sepsis, but invasive Hib disease has been dramatically reduced in countries that have incorporated Hib conjugate vaccines into immunization schedules (Peltola, 2000). In the post Hib vaccine era, NTHi and non-Hib serotypes have continued to cause invasive disease, as indicated by reports from both the United States (Soeters et al., 2018) and Europe (van Wessel et al., 2011; Whittaker et al., 2017). While Hif has remained the predominant encapsulated serotype causing invasive disease in the United States (Soeters et al., 2018), there have been reports indicating an increase of invasive Hia disease, particularly in North America (Bender et al., 2010; Soeters et al., 2018) and in indigenous populations (Kelly et al., 2011; Bruce et al., 2013; Tsang et al., 2014). Additionally, Hia infection and disease have been reported in other parts of the world, including in some European countries (Giufrè et al., 2017; Deghmane et al., 2019; Heliodoro et al., 2020). As a result, efforts have been undertaken to develop a vaccine targeting Hia (Cox et al., 2017).

The Hia capsule is known to be a major virulence factor, and its structure has been previously well characterized (Ulanova and Tsang, 2014; Potts et al., 2019). The capsule locus contains three regions, regions I and III are shared across the six encapsulated serotypes, and region II contains genes unique to the serotype. Some strains of Hia harbor two copies of the capsule locus, with one copy containing a partial deletion in the *bexA* gene (Kroll et al., 1994). This mutation has been reported to increase production of the capsule polysaccharide and increase virulence (Kroll et al., 1993). Another major virulence factor is the IgA protease; this protein plays a key role in disrupting the defense of the mucosa conferred by human IgA1 and enabling *H. influenzae* to colonize the respiratory tract (Kilian et al., 1996). Two types of IgA proteases have been previously reported, with each type being associated with certain serotypes: type 1 are produced by serotypes a, b, d, and f, while type 2 are produced by serotypes c and e (Mulks et al., 1982). Other key virulence factors in *H. influenzae* include genes involved in adhesion (Guerina et al., 1982; van Ham et al., 1994), iron uptake, immune evasion, and endotoxins (High et al., 2015).

Treatment for *H. influenzae* meningitis typically consists of a third generation cephalosporin such as cefotaxime or ceftriaxone (Kimberlin et al., 2018), or ampicillin after antibiotic susceptibility testing of the isolate. Certain genetic markers in *H. influenzae* have been previously reported to confer resistance to β-lactam antibiotics, including the expression of the bla_*TEM*_-1 or bla_*ROB*_-1 β-lactamases (Doern et al., 1997; Hasegawa et al., 2003) and amino acid variations in the penicillin-binding protein 3 (PBP3, encoded by *ftsI*) (Ubukata et al., 2001; Deghmane et al., 2019) which reduce the affinity of this protein to bind to β–lactams such as cephalosporins.

The increase of Hia in North America as a cause of invasive *H. influenzae* disease along with its recent detection in other parts of the world supports the need to better understand the global distribution of this pathogen. We therefore sought to perform a genomic analysis of Hia strains collected from multiple countries in order to identify the major strains in circulation and the factors contributing to their virulence and antimicrobial resistance. Here, we describe the phylogenetic structure of Hia, including placing Hia in the phylogenetic context of other encapsulated serotypes and identifying which Hia strains are circulating in various regions. Additionally, we performed comparative genomics across the Hia strains to identify genetic differences in virulence genes and genes involved in antimicrobial resistance. Finally, we assessed associations between genotype of the Hia strain and clinical disease phenotype and age group.

## Materials and Methods

### Isolate Collection and Whole-Genome Sequencing

The whole genome sequences for 410 invasive Hia isolates were collected from 12 countries, including the United States, Canada, France, Germany, England, Portugal, Spain, Netherlands, Norway, Italy, Poland and Ireland. The isolate counts, time-period and metadata were collected from each participating country and are described in Table 1. The isolates sequenced in this study were collected between 1998 and 2019. All isolates were sequenced using Illumina technologies (HiSeq 2500 or MiSeq) with the exception of four isolates from the United States which were sequenced using PacBio as previously described (Retchless et al., 2016) and one isolate from Italy sequenced using Ion Torrent technology (ThermoFisher Scientific). All sequence reads were transferred to the Bacterial Meningitis Lab at the Centers for Disease Control and Prevention for assembly and genomic analysis. The non-PacBio sequence reads for each sample were trimmed using Cutadapt (Martin, 2011) and subsequently mapped against the human reference genome hg38 as well as a collection of common contaminants and any matching sequence reads were removed. The final sequence reads were used to generate a *de novo* assembly using SPAdes 3.7.0 (Bankevich et al., 2012). Following assembly, contigs with a depth of coverage of less than one-tenth the genome-wide coverage were marked as spurious and removed. Each assembly underwent quality control by checking for the expected genome assembly size of 1.8–2.0 mb along with a minimum of 20× average coverage across the genome to ensure confidence for the subsequent genomic analyses. The sequence data for the Hia isolates used in the project are provided under NCBI Bioproject PRJNA766550.

**TABLE 1:** Sequences received and metadata used in the study.

Country	Sequenced received	Time period	Clinical presentation	Age group
United States	284	1998–2019	Yes	Yes
Canada	30	2006–2011		Yes
France	24	2010–2019	Yes	Yes
Germany	20	2008–2019	Yes	Yes
England	18	2008–2018	Yes	Yes
Portugal	11	1999–2019	Yes	Yes
Spain	8	2014–2019	Yes	
Netherlands	5	2014–2018	Yes	Yes
Norway	3	2017–2019	Yes	Yes
Italy	3	2015–2018	Yes	Yes
Poland	2	2017	Yes	Yes
Ireland	2	2018–2019	Yes	Yes

### Genomic Analysis

Each genome assembly was annotated using BLAST (Altschul et al., 1997) against a custom database consisting of the PubMLST *Haemophilus influenzae* allele collection (Jolley and Maiden, 2010) supplemented with additional loci for manually curated genes involved in antimicrobial resistance. The ORFs for gene sequences in each assembly were confirmed with by two gene prediction software, Prodigal (Hyatt et al., 2010) and GeneMarkS2 (Lomsadze et al., 2018). The MLST schemes from PubMLST (Jolley and Maiden, 2010) were used to identify the sequence type (ST) of each *H. influenzae* isolate. Additional sequences for invasive non-Hia isolates collected between 1998 and 2019 from the United States were included for the phylogenetic analysis and comprised of the following: 109 Hif, 78 Hie, 44 Hib, 6 Hic, and 5 Hid. The phylogenetic analyses comprising Hia genomes and non-Hia genomes were performed using kSNP v3 (Gardner et al., 2015) and annotated using the Interactive Tree of Life platform (Letunic and Bork, 2016). The protein specific phylogenetic trees were generated by aligning protein sequences using clustal omega (Sievers et al., 2011) and using the subsequent alignment to create a maximum-likelihood phylogenetic tree using RAxML (Stamatakis, 2014). The Single Nucleotide Polymorphism (SNP) difference calculations were performed using custom python scripts with the SNP alignment generated by kSNP as input. Genes involved in virulence were identified using the Virulence Factor Database platform (Liu et al., 2018). For the comparative genomics analyses, custom python scripts were used to create spreadsheets looking at gene presence or absence across clades, identify alleles of genes harboring internal stop codons within specific clades, and to characterize the variant present in antimicrobial resistance genes. The genome-wide association study was performed using the PySeer (Lees et al., 2018) package on 319 invasive Hia isolates for which clinical presentation data was provided. The presence or absence of the capsule duplication was detected by mapping the sequence reads for isolates sequenced using Illumina technologies against their overall genome assembly and only against the capsule region and calculating the difference in average depth of coverage.

### Statistical Analysis

The hypergeometric enrichment test was used to calculate significant over-enrichment of major sequence types observed in the study within age groups; this was limited to sequence types that contained at least 30 isolates to ensure sufficient sample size for statistical confidence.

## Results

### The Hia Isolates Formed Four Major Clades

**FIGURE 1:**
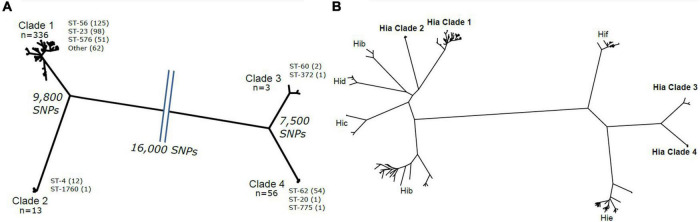
**(A)** Phylogeny showing the phylogenetic structure of the Hia isolates. Each clade is marked with the number of isolates and the major sequence types and their counts. The SNP difference between each clade is shown. **(B)** The phylogeny is shown including all Hia isolates and the additional encapsulated serotypes. The four Hia clades are shown in bold.

Among the 410 Hia isolate genome sequences obtained for the study, 76.59% (314/410) were obtained from North American countries, while 23.41% (96/410) were obtained from European countries. The two isolate sequences from Ireland failed our quality control checks and were therefore excluded from further genomic analysis. Phylogenetic analysis revealed that the remaining 408 Hia isolates formed four major clades (Figure 1A) consisting of the following: clade 1 (*n* = 336), clade 2 (*n* = 13), clade 3 (*n* = 3) and clade 4 (*n* = 56). Each of the four clades contained isolates from both North America and Europe; specifically, clade 1 contained similar percentages of isolates from North America and Europe (80.57 and 88.30%, respectively), clades 2 and 3 contained higher percentages of European isolates (8.51 to 1.59%, and 2.13 to 0.32%, respectively), and clade 4 contained a higher percentage of North American isolates (17.52 to 1.06%) (Supplementary Material 1). These clades were genetically diverse from one another, with clades 1 and 2 differing by an average of 9,800 SNPs, clades 3 and 4 differing by an average of 7,500 SNPs, and clades 1 and 2 differing from clades 3 and 4 by an average of 16,000 SNPs. Each of these major clades contained one or more predominant sequence types, with ST-56 (*n* = 125), ST-23 (*n* = 98) and ST-576 (*n* = 51), comprising 81.50% (274/336) of clade 1; ST-4 (*n* = 12) comprising 92.31% (12/13) of clade 2, ST-60 (*n* = 2) comprising 67% (2/3) of clade 3, and ST-62 (*n* = 54) comprising 96.40% (54/56) of clade 4. The Hia isolate genomes were compared against other encapsulated serotypes, including Hib, Hic, Hid, Hif and Hie (Figure 1B). A clear delineation was observed with Hia clades 1 and 2 being more closely related to Hib, Hid and Hic, while Hia clades 3 and 4 were more closely related to Hif and Hie.

### Most Hia Isolates Belonged to Clade 1

**FIGURE 2:**
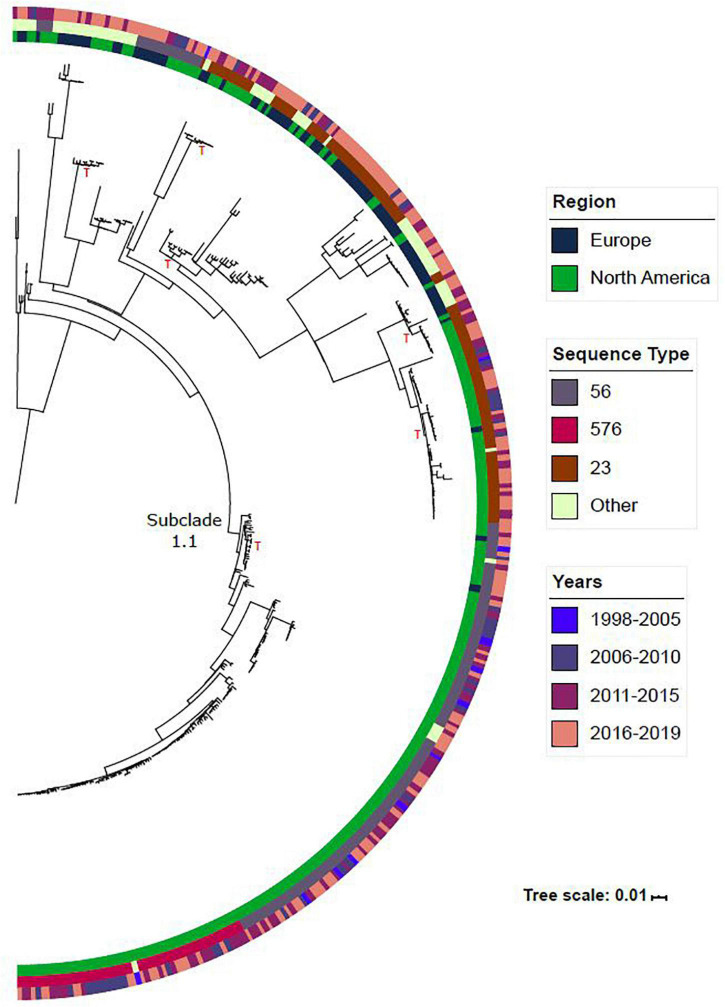
The phylogeny for clade 1 is shown in this figure. Metadata such as region, sequence type and years are included for reference. Other sequence type refers to STs with less than 15 isolates. A red “T” is included next to subclades that contain isolates from both North America and Europe. Subclade 1.1 is labeled in the figure.

The vast majority (82.3%; 336/408) of the Hia collection belonged to clade 1. The phylogeny for this clade is shown in Figure 2. While this clade was diverse with a total of 24 unique STs identified, 16 STs differed by only one MLST allele to one of the three major STs identified in this clade (ST-56, ST-23 and ST-576), with the remaining 5 differing by two MLST alleles. Among the major STs identified within this clade, ST-56 was predominantly found among North American isolates (90.4%; 113/125), while ST-23 was found across isolates collected from both North America (65.3%; 64/98) and Europe (34%, 34/99). Across each of these sequence types, subclades were formed within the tree containing isolates from both Europe and North America, indicating the circulation of these strains across both regions. A low-diversity subclade was formed within this phylogeny and has been labeled as subclade 1.1. The isolates belonging to subclade 1.1 differ by an average of 115 SNPs, as compared to clade 1 overall which differs by an average of 1,300 SNPs. The vast majority of the isolates in subclade 1.1 were obtained from North America (98.1%; 160/163). ST-576 was entirely found within this subclade and was only found among North American isolates. As shown in Supplementary Material 2, the clade harboring the ST-576 isolates shares an ancestral lineage with ST-56 and diverged from a most recent common ancestor with ST-56. The earliest ST-576 isolate in our collection was collected in 2006, indicating that ST-576 diverged from ST-56 prior to 2006, and that this strain has been in circulation in North America since.

### Genetic Differences in Major Virulence Factors Were Identified Among the Four Clades

**FIGURE 3:**
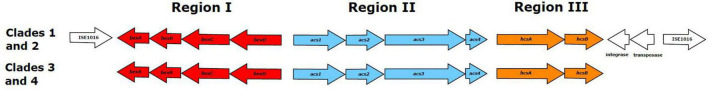
The capsule regions for Hia isolates belonging to each of the four clades is shown. Clades 1 and 2 contain genes encoding an integrase and transposase proteins and are flanked by IS1016 elements.

**FIGURE 4:**
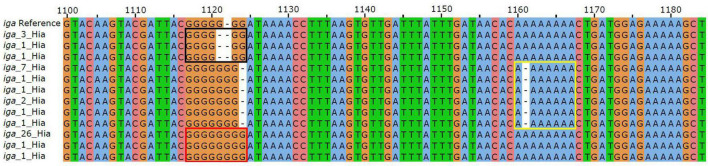
Sequence alignment of the *iga gene* consisting of regions 1,100–1,180 with respect to the reference sequence. The three sites of indels are boxed in black, yellow and red. Each variant is labeled with the count of isolates harboring that variant.

All Hia isolates were confirmed to contain the genes encoding the serotype a capsule locus. There were genetic differences identified within the capsule locus among the clades. Most notably, the capsule locus in clades 1 and 2 were flanked by IS1016 and contained two genes encoding a transposase and integrase downstream of *hcsB* that were not present in clades 3 and 4 (Figure 3). The presence of the previously reported capsule duplication in Hib was checked in the Hia isolates. As most isolates were sequenced using short read WGS methodologies, the capsule duplication would not be evident in the resulting genome assemblies. As such, a read mapping approach was undertaken to confirm the presence of the capsule duplication in the isolates (Supplementary Material 3). All Hia isolates belonging to clades 3 and 4 had similar average depth of coverage of the capsule region as compared to the rest of the overall genome (0.8–1.2× fold difference), indicating that the capsule duplication was likely not present in these isolates. Conversely, the vast majority of isolates belonging to clades 1 and 2 (74.6%; 259/347) had 1.5× or greater average depth of coverage in the capsule region as compared to the entire genome (1.5×–7.2× fold difference), indicating that the capsule duplication is likely present in these isolates. In addition to the capsule locus, the Hia collection was scanned for the sequences of 90 known *H. influenzae* virulence factors as defined by the Virulence Factor DataBase (Liu et al., 2018). Information about the virulence genes identified is provided in Supplementary Material 4. The following seven virulence factors were absent from all isolates: HAEM0992 (hypothetical protein), *kfiC*, *orfE*, *siaA*, *wbaP*, *rffG* and *lex2B*. The *hifABCDE* genes were only present in clades 3 and 4, with all isolates in these clades harboring the genes. All isolates in clade 4 (*n* = 56) contained a disrupted *pilB* gene harboring a point mutation at position 682 C- > T creating a premature internal stop codon. Two distinct *hgpC* genes were identified in 165 isolates belonging to clade 1, with 100 of these belonging to subclade 1.1. The *iga gene* was identified in all Hia isolates but was disrupted in 10.2% (42/408) of the collection. Three distinct indel mutations introducing frameshifts were identified comprising 73% (30/42) of the isolates with disrupted *iga gene*s. Each of these indel mutations are represented in Figure 4. These mutations consist of the following: 1121delG (black box), 1122insG (red box) and 1161delA (yellow box). The remaining 27% (17/63) of the disrupted *iga gene*s consisted of various point mutations and indels with no distinct pattern. Nearly all isolates belonging to clade 1 and subclade 1.1 (99.4%; 334/336) harbored the outer membrane protein P5 allele 1, while the isolates from other clades harbored different alleles for the P5 protein.

### Genetic Elements Associated With Antimicrobial Resistance of Hia

The Hia genome collection was scanned for the presence of genes known to confer resistance to antimicrobials. The *cat* gene was identified in 1.7% (7/408) of the isolates in the collection. All 7 of these isolates belonged to clade 2, ST-4 and were collected from the following countries: United States (*n* = 3), France (*n* = 1), England (*n* = 1), Italy (*n* = 1) and Netherlands (*n* = 1). The bla*_*TEM*_-1* gene was identified in 1.7% (7/408) of the isolate collection. These isolates belonged to the following clades: clade 1 (*n* = 2), clade 2 (*n* = 3) and clade 4 (*n* = 2), and were collected from the following countries: United States (*n* = 4), England (*n* = 2) and Canada (*n* = 1). The bla*_*ROB*_-1* gene was not identified in any of the Hia isolates.

**FIGURE 5:**
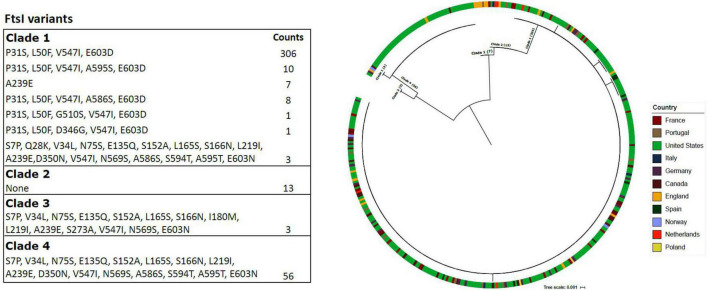
The PBP3 variants are shown in the table for each of the major Hia clades. The phylogeny consists of PBP3 protein sequences and is annotated with the respective country of the isolate. The branches include which of the four major Hia clades the isolates belong to.

The diversity of the penicillin-binding protein 3 (PBP3) was assessed across the Hia isolate collection (Figure 5). There was a predominant PBP3 variant for each of the major clades. The majority of clade 1 isolates (91%; 306/336) harbored the variant containing amino acid substitutions P31S, L50F, V547I and E603D. None of the clade 2 isolates harbored a variant that was different than the reference. Only one PBP3 variant was detected within clades 3 and 4. Seven European isolates within clade 1 harbored a distinct PBP3 variant containing only amino acid substitution A239E; these isolates consisted of six from England and one from Portugal. Additionally, there were three clade 1 isolates that harbored a PBP3 variant that was more closely related to the main variant in clade 4, and these were collected from the following countries: Norway (*n* = 1), United States (*n* = 1) and Canada (*n* = 1). Finally, three isolates from clade 1, as well as the isolates of clade 4, harbored the D350N substitution that was previously reported in beta-lactamase negative ampicillin resistant (BLNAR) isolates (Deghmane et al., 2019), but has not been reported to confer resistance on its own. However, no other BLNAR-linked substitutions were observed among Hia isolates of this study (Figure 5). Furthermore, none of the isolates harbored either of the BLNAR defining N526K or R517H substitutions.

### Genetic Association With Clinical and Epidemiological Characteristics

We obtained clinical presentation data for 319 Hia isolates in the collection. These clinical presentations were as follows: 101 pneumonia, 85 meningitis, 73 bacteremia, 24 septicemia, 20 other infections, 6 septicemia and meningitis, 5 arthritis, 4 epiglottitis, and 1 osteomyelitis. A genome wide association study was conducted on these 319 Hia genomes in order to identify regions of the DNA that may be significantly associated with the clinical presentation. Both k-mers and SNPs were considered in this analysis. This analysis revealed a low heritability (h^2^) estimate of 0.1, indicating that very little of the phenotypic variation was due to the genetic variation present in this population. Additionally, no k-mers or SNPs were identified to be significantly associated with any of the given clinical phenotypes.

Age group data was available for a total of 336 Hia isolates. These age groups were as follows: < 1, 1–4, 5–10, 11–17, 18–34, 35–49, 50–64 and > 65. Age groups 5–10, 11–17, and 18–34 were merged into one age group (5–34) to provide sufficient sampling for the analysis. A hypergeometric test for enrichment was performed to determine if any of the major sequence types in our study were significantly over-enriched within any of these age groups (Supplementary Material 5). The following sequence types with 30 or more isolates that had age group data were included in this analysis: ST-56 (*n* = 109), ST-23 (*n* = 77), ST-62 (*n* = 46) and ST-576 (*n* = 34) for a total of 266 isolates. No sequence type was identified to be significantly over-enriched within any of the tested age groups.

## Discussion

In this study, the genomes for Hia isolates collected from 12 countries were compared in order to better understand the genomic factors of this rising cause of *H. influenzae* disease. We identified four major clades that were distinct from each other and from other encapsulated *H. influenzae* serotypes. Most isolates belonged to clade 1, suggesting that the isolates belonging to clade 1 are the major cause of Hia disease. It would be interesting to further investigate whether the strains belonging to clade 1 are more virulent or transmissible compared to strains of the other Hia clades. The predominant sequence types in clade 1 were ST-56, ST-23 and ST-576. ST-576 was identified in a low diversity subclade within clade 1 and only contained isolates from North America. ST-576 shares an ancestral lineage with ST-56 and diverged from a most recent common ancestor of ST-56 sometime prior to 2006. Obtaining more Hia isolates from other countries would be needed to confirm if this subclade 1.1 is exclusively circulating within North America or in other countries as well.

Previously, two distinct Hia clades have been reported (Potts et al., 2019) which correspond to clades 1 and 4 in our study. The genome sequences belonging to clades 3 and 4 became available more recently, providing additional resolution on the phylogenetic structure of this serotype. While the presence of four major clades suggests that Hia may be more diverse than other encapsulated serotypes, including additional encapsulated *H. influenzae* isolates in our comparison would be needed to confirm this result. Hib contained two distinct clades and was the only other serotype to contain more than one phylogenetically distinct group.

Our collection was largely dominated by isolates collected from North America. This could be explained by the fact that Hia is much less prevalent in Europe as compared to North America (Whittaker et al., 2017), suggesting that our sampling is likely representative of the Hia disease burden in these countries for recent years. Furthermore, we did identify isolates from both North America and Europe in each of the four major clades, as well as several instances of subclades containing isolates from both North America and Europe, indicating that these strains have been transmitted across these regions.

It has been observed previously that Hia can cause severe disease similar to Hib (Ulanova and Tsang, 2014; Plumb et al., 2018) in the pre-Hib vaccination era. In our study, we checked for virulence genes that may have been shared among Hia and Hib which could explain this similarity in disease severity but did not identify anything remarkable. We also examined our Hia isolate collection for the presence of the duplicated capsule locus, which has been previously reported to be associated with increased virulence in both Hia and Hib, and found evidence of this mutation being present in the vast majority of the Hia isolates belonging to clades 1 and 2 and absent in the clade 3 and 4 isolates. This finding is supported by the isolates in Hia clades 1 and 2 sharing the same lineage as Hib. Furthermore, we showed that the most predominant Hia strains in circulation, those belonging to clade 1, were most closely related genetically to Hib than the other serotypes. Additionally, it has been previously shown that the Hia and Hib genomic capsular region share stark similarities (Potts et al., 2019), specifically in region II, which encodes biosynthesis genes unique to each serotype. Among these, *acs1* (Hia) and *bcs1* (Hib) are highly similar, sharing a minimum of 96.13% nucleotide sequence similarity (Potts et al., 2019). These factors, including the presence of the capsule duplication and the overall higher level of genetic similarity across the genome and in the capsule region could explain the similar levels of disease severity among Hia and Hib.

Among the four Hia clades, the capsule region of isolates belonging to clades 1 and 2 were flanked by insertion sequence IS1016 and additionally contained two genes encoding a transposase and an integrase. This same pattern of capsule being flanked by IS1016 and containing a transposase and integrase gene was also observed in Hib, Hic and Hid, and seems to be a trait shared among all serotypes in that lineage. Conversely, this pattern was absent from Hia clades 3 and 4, Hif and Hie. This finding is consistent with previous literature describing two phylogenetically distinct divisions of encapsulated *H. influenzae*, one containing capsule regions flanked by IS1016, and the other with IS1016 being absent from the capsule region (Musser et al., 1988; Satola et al., 2008). The presence of a transposase and integrase within the IS1016 flanked region of the capsule of these genomes suggests that these strains likely acquired the capsule at some point and could be an additional factor in the similar level of disease severity among Hib and Hia.

A total of 90 virulence genes were examined for notable differences between the four major Hia clades. These genes spanned several different classes of virulence factors, such as adherence, endotoxin, immune evasion and iron uptake. Seven of the virulence genes involved in the lipooligosaccharide (LOS) endotoxin biosynthesis were absent from all Hia isolates: HAEM0992 (hypothetical protein), *kfiC*, *orfE*, *siaA*, *wbaP*, *rffG* and *lex2B.* Four of these, *kfiC*, *orfE*, *siaA* and *wbaP*, have been reported previously to be absent in Hia, as well as in other serotypes (Pinto et al., 2019). The pilus genes *pilABCD* were present across all of the four Hia clades, with all isolates in clade 4 harboring a disrupted *pilB* with the same point mutation introducing a premature internal stop. These four genes are required for biogenesis and function of the type IV pilus (Tfp), and each gene has been shown to be required for pilus function (Carruthers et al., 2012). Further experiments would be required to confirm the lack of expression of the PilB protein in clade 4 isolates as a result of this mutation and subsequently assess the potential impact on the virulence of these strains. All Hia isolates across the four clades contained both the *hgpB* and *hgpC* gene, with many isolates in clade 1 harboring two distinct *hgpC* genes. Two other *hgp* genes have been described in the literature, *hgpA* (Morton et al., 1999) and *hgpD* (Harrison et al., 2005), but we did not identify any Hia isolates harboring either of these genes in our collection. The *iga gene* was identified in all Hia isolates, with a small fraction of the isolates (14%) containing genes harboring disrupted ORFs; these disruptions were most commonly introduced by indels in guanine or adenine simple sequence repeat (SSR) regions resulting in frameshift mutations. SSRs serve as an important mechanism for adaption in *H. influenzae* across many genes (Power et al., 2009), and similar mutations in *iga* genes have been previously described in strains of NTHi collected from the airways of patients with Chronic Obstructive Pulmonary Disease (Gallo et al., 2018). Almost all isolates in clade 1 and subclade 1.1 shared an exclusive allele (allele 1) encoding the outer membrane protein P5, while the isolates from other clades harbored different P5 alleles. These different P5 alleles primarily vary by four outer surface loops that are likely involved in the capacity of binding the complement negative regulator, factor H, which is an important inhibitor of alternative pathway activation (Rosadini et al., 2014).

The rise of mutations reported to confer resistance to β-lactam antibiotics is of concern, particularly since this antibiotic class is a primary treatment for *H. influenzae* meningitis (Kimberlin et al., 2018). In general, the presence of known factors contributing to β-lactam resistance were low in our collection, as the bla*_*ROB*–1_* gene was not identified in any of the Hia isolates, and the bla*_*TEM*–1_* gene was only identified in 7 Hia isolates. While we did identify a primary PBP3 variant for each of the four Hia clades, there was genetic variation present in several isolates that was based on region. None of the variants identified have been previously reported to confer resistance, and additional antibiotic susceptibility would be necessary to determine the effects of these specific mutations.

The mechanisms for invasion of *H. influenzae* into the respiratory tract and bloodstream have been previously well described (Clementi and Murphy, 2011; King, 2012) and is dependent on a variety of factors, including those within the host and those within the bacteria. In our study, we did not identify any regions of the Hia genomes to be significantly associated with any of the given clinical disease phenotypes, suggesting that the clinical disease manifestation is a likely a result of the host’s response to the pathogen. Among the predominant Hia sequence types in our study, none were significantly more frequent in any age groups considered; however, the highest percentage of the Hia isolates with age data in our study were collected from patients below the age of 4 (150/336; 45%), and the second highest was from patients above the age of 50 (125/336; 37%).

## Conclusion

In the post-Hib vaccination era, strains of NTHi and encapsulated non-b serotypes have continued to be major causes of *H. influenzae* disease. Among these, Hia is of particular interest given increases in cases reported in North America and in indigenous populations. The results of our study have shown that the reported cases of Hia in North America and Europe in the past two decades have belonged to four genetically distinct clades, with ST-56, ST-23, ST-576, and ST-4 being the predominant sequence types. Variations in virulence genes, such as mutations disrupting the ORFs, as well as the presence/absence and variation of antimicrobial resistance markers were identified within the clades. Additional tests of virulence and antibiotic susceptibility would be needed to determine the impact of these variations among the isolates.

## Data Availability Statement

The datasets presented in this study can be found in online repositories. The names of the repository/repositories and accession number(s) can be found in the article/Supplementary Material.

## Author Contributions

NT, M-KT, and XW contributed to the study design, data collection, analysis, and critical review and approval of the manuscript. NT prepared the first manuscript draft. All authors have contributed to data collection, investigation, and critical review and approval of the manuscript.

## Author Disclaimer

The findings and conclusions in this report are those of the authors and do not necessarily represent the official position of the Centers for Disease Control and Prevention.

## Conflict of Interest

The authors declare that the research was conducted in the absence of any commercial or financial relationships that could be construed as a potential conflict of interest.

## Publisher’s Note

All claims expressed in this article are solely those of the authors and do not necessarily represent those of their affiliated organizations, or those of the publisher, the editors and the reviewers. Any product that may be evaluated in this article, or claim that may be made by its manufacturer, is not guaranteed or endorsed by the publisher.
