# Supplementary figures and images for: Phylogenetic Structure and Comparative Genomics of Multi-National Invasive Haemophilus influenzae Serotype a Isolates
Source: Front Microbiol. 2022 Mar 24;13:856884. doi: 10.3389/fmicb.2022.856884 (PMC8988223; doi:10.3389/fmicb.2022.856884)

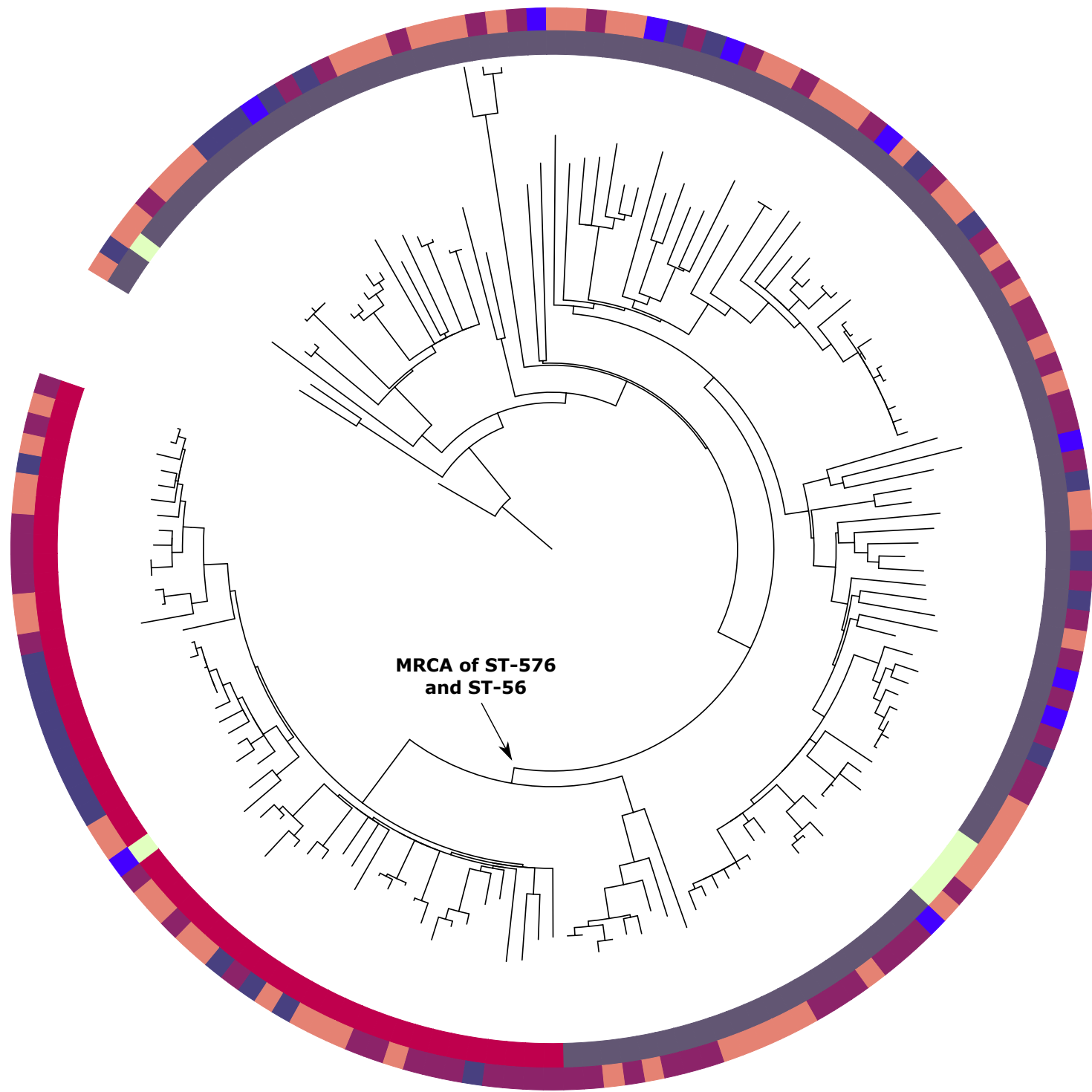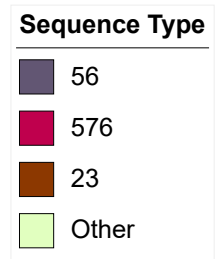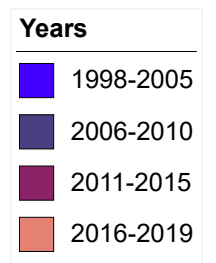

MRCA of ST-576  
and ST-56

Tree scale: 0.01

Supplement: Supplementary file 1 [file Data_Sheet_1.PDF]
