# Supplementary material for: Phylogenetic Structure and Comparative Genomics of Multi-National Invasive Haemophilus influenzae Serotype a Isolates
Source: Front Microbiol. 2022 Mar 24;13:856884. doi: 10.3389/fmicb.2022.856884 (PMC8988223; doi:10.3389/fmicb.2022.856884)
Supplement: Supplementary file 2 [file Data_Sheet_2.DOCX]

**
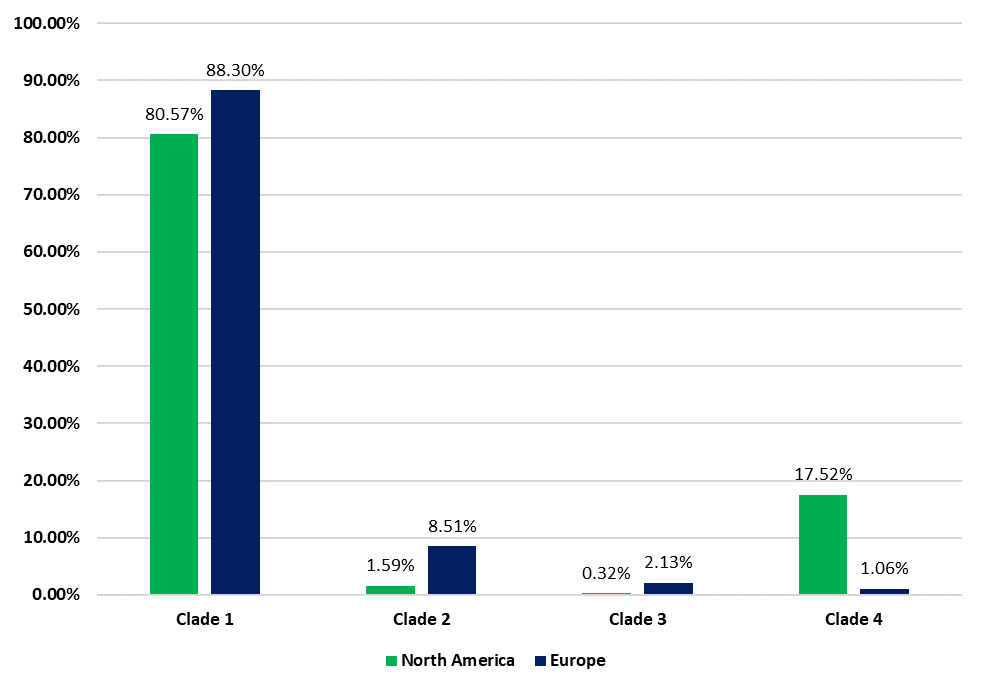
Percentage of isolates from each region that belonged to each of the four major clades of *Haemophilus influenzae* serotype a**
