# Supplementary material for: Phylogenetic Structure and Comparative Genomics of Multi-National Invasive Haemophilus influenzae Serotype a Isolates
Source: Front Microbiol. 2022 Mar 24;13:856884. doi: 10.3389/fmicb.2022.856884 (PMC8988223; doi:10.3389/fmicb.2022.856884)
Supplement: Supplementary file 5 [file Data_Sheet_5.DOCX]

| **Sequence Types (# isolates)** | **Age Groups (# isolates)** | | | | | |
| --- | --- | --- | --- | --- | --- | --- |
|  | **<1**  **(n=67)** | **1-4 (n=57)** | **5-34 (n=21)** | **35-49 (n=19)** | **50-64 (n=55)** | **>65 (n=47)** |
| **ST-56 (n=109)** | 0.801568529 | 0.712352118 | 0.091296123 | 0.202454109 | 0.502424598 | 0.816078099 |
| **ST-23 (n=77)** | 0.815759756 | 0.161325133 | 0.781904883 | 0.48703743 | 0.874320617 | 0.24822332 |
| **ST-62 (n=46)** | 0.234448212 | 0.823303578 | 0.909219455 | 0.976464131 | 0.338342613 | 0.273383103 |
| **ST-576 (n=34)** | 0.203936317 | 0.626259868 | 0.781321542 | 0.729272638 | 0.403413395 | 0.890961151 |

**Hypergeometric p value (P (X >= x) for over-enrichment of the major sequence types of the study for each age group.**
